# Current clinical practice of electroconvulsive therapy and repetitive transcranial magnetic stimulation in psychiatry, a German sample

**DOI:** 10.1007/s00406-020-01099-x

**Published:** 2020-01-29

**Authors:** Charles Timäus, Jonathan Vogelgsang, Bernhard Kis, Katrin Radenbach, Claus Wolff-Menzler, Kiriaki Mavridou, Stephan Gyßer, Philipp Hessmann, Jens Wiltfang

**Affiliations:** 1 Department of Psychiatry and Psychotherapy, University Medical Center Göttingen, University of Göttingen, Von-Siebold-Str. 5, 37075 Göttingen, Germany; 2 Division of Software Development and Business Intelligence, GSG Consulting GmbH, Flughafenring 2, 44319 Dortmund, Germany; 3 German Center for Neurodegenerative Diseases (DZNE), Von-Siebold-Str. 3a, 37075 Göttingen, Germany; 4 iBiMED, Medical Science Department, University of Aveiro, Aveiro, Portugal; 5 Department of Psychiatry, Psychotherapy and Psychosomatics, St. Elisabeth Hospital Niederwenigern, Contilia Group, Essener Straße 31, 45529 Hattingen, Germany

**Keywords:** rTMS, ECT, Ambulatory, Neurostimulation, Brain stimulation, Noninvasive

## Abstract

The purpose of the study was to evaluate the current clinical practice of Electroconvulsive Therapy and Repetitive Transcranial Magnetic Stimulation in German psychiatry. Case-based data (> 1.000.000 cases) were collected according to §21 of the German hospital remuneration law from January 2015 to December 2017. The study cohort comprises approximately 35–40% of the annual psychiatric cases and hospitals in Germany. Frequency of ECT and rTMS cases were investigated considering main diagnoses according to ICD-10 and treatment settings (inpatient vs. day-care). ECT cases with short-term hospitalization (≤ 4 days) were supposed to be maintenance ECT cases. A linear regression analysis was conducted to estimate trends in the use of ECT and rTMS. Different groups were compared using Chi-square tests. ECT and rTMS cases appear to increase in total during the observation period possibly due to facilities newly introducing ECT and rTMS but also to increased frequency of treatments. Both treatments were rarely performed in day-care settings (0.89% and 11.25%). ECT was performed in 1.72% of all cases with affective disorders and in 1.48% with major depressions, respectively. Age ≥ 65 years, females, severe and psychotic depression were significantly associated with a higher rate of ECT cases. > 40% of all ECT cases were possibly maintenance ECT cases. Only 0.60% of these were performed in day- care settings. rTMS was primarily performed in major depression (86,7% of all rTMS cases). This study suggests a growing demand for ECT and rTMS. Nevertheless, the use of ECT is still low compared to the high prevalence of treatment resistant depression. The use of rTMS is even lower and seems to be restricted to specialized institutions. Maintenance ECT is frequently carried out in an inpatient setting. Limitations of this study are the case- and group-based analysis, missing data on outpatient services and treatment sessions per case. Therefore, the database is not necessarily representative for the entire German healthcare system. Further studies are needed to verify the presented findings and should address the feasibility of ambulatory and day-care ECT services.

## Introduction

Two noninvasive stimulation methods, rTMS (repetitive transcranial magnetic stimulation) and ECT (electroconvulsive therapy), are effective therapeutic options for diverse psychiatric disorders, i.e. unipolar and bipolar depression [1–5] and psychotic disorders [6, 7]. Several guidelines give evidence-based recommendations regarding indications, application and safety aspects of ECT and rTMS [8–10]. Recently an evidence-based guideline on the use of a third technique, transcranial direct current stimulation, was published, which induces neuroplasticity by a transcranial electrical stimulation at subthreshold intensities [11–14]. ECT is considered as the most effective treatment for severe major depression and is superior to the effects of antidepressant drugs [5]. Moreover, ECT seems to be superior to rTMS, especially considering psychotic depression, and has proved high efficacy in the elderly [15–17]. However, ECT is still suffering from low acceptance compared with less invasive treatment options for psychiatric diseases (e.g. psychotherapy) [18]. rTMS has been approved for major depression [19] and recently for obsessive compulsive disorder [20]. Accessibility to rTMS is still restricted to a limited number of healthcare institutions and the technique is usually carried out within clinical studies. Since rTMS is not generally eligible for reimbursement in Germany, patients often have to pay for rTMS.

Therapeutic alternatives, such as rTMS and ECT, are urgently required since treatment resistance in psychiatric disorders is very frequent. For instance, it was shown that approximately 30% of depressed patients present with treatment resistant depression [21, 22]. The German guideline strongly recommend ECT if the current depressive episode do not adequately respond to at least two trials of antidepressant pharmacotherapy. Additionally, rTMS can be an option if at least one antidepressant medication was not successful [23].

To our knowledge, a systematic up-to-date description of the application of rTMS and ECT in German psychiatric clinics is missing [24–26]. In the present study, we conducted a descriptive analysis of the clinical use of rTMS and ECT in the German mental healthcare system. Therefore, a case-based dataset comprising the observation period from January 2015 to December 2017 was analyzed.

## Methods

Data were collected from the GSG-Benchmarking Project, which was conducted by GSG-Consulting Germany (PEPP-Benchmarking, data storage and access provided by GSG-Consulting). All data were primarily documented by the healthcare providers for cost reimbursement by the healthcare insurance companies and collected from participating hospitals and departments in Germany. In Germany, routine data of psychiatric inpatient care are collected on a basis of a special reimbursement system which is called “PEPP”. 184 psychiatric hospitals in Germany provided data for this project in accordance with §21 of the German hospital remuneration law (“Krankenhausentgeltgesetz”). We analyzed data between January 2015 and December 2017. Inclusion criterion was a minimum age of 18. The above-mentioned measures (rTMS and ECT) were identified according to the German procedure classifications (OPS, “Operationen- und Prozedurenschlüssel”). The codes for rTMS and ECT were 8-632 and 8-630, respectively. Frequencies of rTMS and ECT cases in inpatient and day- care settings were assessed. Moreover, we analyzed the patients’ main diagnoses at the time of discharge, gender and age. The diagnoses based on the German International Statistical Classification of Diseases and Related Health Problems version 10 (ICD-10) as hospitals in Germany are instructed by the statutory health insurance to code a diagnosis according to ICD-10. There are usually two forms of ECT treatments. Acute ECT treatments intend to induce improvement of clinical symptoms and maintenance ECT treatments aim to prevent relapses of clinical symptoms. Due to the case- based structure of the database neither detailed information on patient´s clinical status nor the total number of ECT treatments and their indications, i.e. maintenance or acute ECT treatments, were directly accessible. In this case study approach, a hospital stay that did not exceed 4 days was supposed to correspond to a hospitalization due to maintenance ECT. A hospital stay longer than 4 days was supposed to indicate an acute phase as it usually comprises 6 to 12 ECT sessions which were usually carried out during 2–4 consecutive weeks.

### Description of the study cohort and demographic characteristics

The entire database of the GSG benchmarking project consists of 1.184.710 cases spanning the period January 2015 to March 2018. Between January 2015 and December 2017, a total of 1.032.094 cases were adults. 1.012.961 cases were attributed to the main diagnoses for mental and behavioural disorders (F00–F99) according to ICD-10 with a mean age of 46.5 ± 17.6 years. 531.939 cases or 52.5% with the main diagnoses F00–F99 were male. Information on gender was not available in 33 cases. Mood disorders (F30–F39) were found with a total of 353.884 cases (35% of the F00–F99 cohort), followed by mental and behavioural disorders due to psychoactive substance use with a total of 288.471 (28.5%) and schizophrenia, schizotypal and delusional disorders (F20–F29) with a total of 151.210 (14.9%). Table 1 provides a detailed description of the present study cohort. The number of psychiatric clinics and the reported number of psychiatric cases were shown on a year to year basis. Additionally, governmental data of the Federal Office of Statistics of Germany (Statistisches Bundesamt, Destatis) on the psychiatric health care system were presented for comparative purposes [27]. 61 psychiatric clinics annually carried out ECT in 2016 and 2017 while 60 clinics performed ECT in 2015. Therefore, knowing that approximately 185 psychiatric clinics provide ECT in Germany, less than one third of all clinics performing ECT contributed data for this study. 10 clinics contributed data on the use of rTMS in 2015 and 2016 while 11 clinics performed rTMS in 2017. The study cohort comprises approximately 35% of the annual psychiatric cases registered by the Federal Office of Statistics of Germany.

**Table 1:** Description of the case study cohort (upper section) and epidemiologic data of the Federal Office of Statistics of Germany (lower section): overall number of included clinics and clinics providing ECT or rTMS; overall number of clinics are also given as proportions (%) of all psychiatric clinics registered by the Federal Office of Statistics of Germany; number of inpatient and day-care cases of the study cohort; total number of cases are also given as proportions (%) of all cases registered by the Federal Office of Statistics of Germany

	2015	2016	2017
Number (%) of clinics study—(all)	171 (41.81)	171 (41.81)	160 (39.31)
Number of clinics study—(ECT)	60	61	61
Number of clinics study—(rTMS)	10	10	11
Number of cases—inpatient	285,243	296,024	277,150
Number of cases—day-care	50,100	53,578	50,866
Number (%) of cases—total	335,343 (34.59)	349,602 (36.35)	328,016 (34.34)
Number of clinics—Germany^a^	409	409	407
Number of cases—inpatient^a^	824,521	816,316	806,227
Number of cases—day-care^a^	145,003	145,574	149,070
Number of cases—total^a^	969,524	961,890	955,297

^a^Statistisches Bundesamt. Grunddaten der Krankenhäuser 2015, 2016, 2017. https://www.destatis.de/DE/Themen/Gesellschaft-Umwelt/Gesundheit/Krankenhaeuser/_inhalt.html#sprg234206. Accessed 7 November 2019

### Statistics

The data were statistically analyzed with Prism GraphPad Version 8. Different groups were compared using Chi-square tests. A Bonferroni correction for multiple comparisons was applied. Considering a total of 33 tests, a significance of results was assumed if *p* was < 0.0015. For estimating the relationship between frequency of ECT or rTMS and time (quarters) a linear regression analysis was conducted. Goodness-of-fit was expressed in *r*^2^. The *p* value was calculated from an *F* test. The value of *F* and its degrees of freedom (DFn, DFd) were given. If the *p* value was < 0.05 it was assumed that the slope was significantly different than zero.

## Results

### Electroconvulsive Therapy

#### Frequency of ECT from January 2015 to December 2017

Considering the main diagnoses F00–F99 and the minimum age criterion, ECT was applied in 8369 cases corresponding to a proportion of 0.83%. ECT was performed in 6102 cases with affective disorders (F30–F39) corresponding to a share of 1.72% and in 4799 cases with major depression (F32 and F33) corresponding to a share of 1.48%. Considering psychotic disorders (F20-F29) a total of 2084 cases were treated with ECT corresponding to a share of 1.38%. ECT was less often performed in day- care settings compared to inpatient settings (75 cases vs. 8294 cases).

**Fig. 1:**
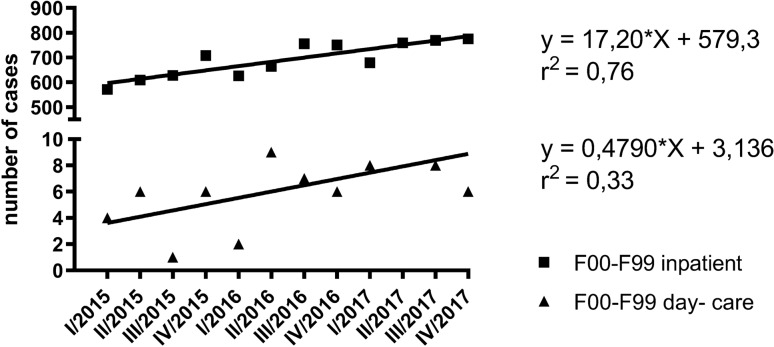
(Left side) Frequency of ECT cases from January 2015 to December 2017 in inpatient and day-care settings considering F00–F99 diagnoses; number of cases (*y* axis) are plotted per quarter (*x *axis); (right side) linear regression analysis: equation of best- fit line, goodness-of-fit expressed in *r*^2^

As shown in Fig. 1, the number of cases in inpatient settings continuously increased each quarterly period from January 2015 to December 2017. The linear regression model suggests a high relationship (*r*^2^ 0.76, *F* 31.05, DFn 1.000, DFd 10.00, *p* < 0.0002) between the variables. Much of the variation of the response variable (cases) can be explained by the predictor (time period). The goodness-of-fit was low in terms of day- care settings (*r*^2^ 0.33, *F* 5.014, DFn 1.000, DFd 10.00, *p* = 0.0491).

#### Frequency of ECT considering main diagnoses from January 2015 to December 2017

The total number of cases treated with ECT appeared to increase from January 2015 to December 2017. This observation was found throughout all affective (F30–F39) and psychotic (F20–F29) disorders. Considering the F00–F99 diagnoses, the relative use of ECT appeared to be higher in 2017 compared to that of 2015 and 2016. This was also true for affective (F30–F39) disorders. Compared to 2015 the relative use of ECT in psychotic (F20–F29) disorders was higher in 2017 as provided in detail in Table 2. It should be taken into account that the statistical analyses could be partly influenced by the smaller case database of the year 2017 and did not necessarily reflect a higher relative use of ECT in 2017 (see Table 1).

**Table 2:** Comparison of the groups with and without ECT between January 2015 and December 2017. Cases with the ICD-10 diagnoses F00–F99 (upper section), F30-F39 (middle section) and F20–F29 (bottom section) were analyzed. Significant results are highlighted in bold characters

Year	ECT	No ECT	Total	ECT (%)		*χ*^2^	*df*	*p* value
F00–F99								
2015	2533	332,810	335,343	0.76	2015 vs. 2016	5.806	1	< 0.0160
2016	2820	346,782	349,602	0.81	2016 vs. 2017	25.24	1	** < 0.0001**
2017	3016	325,000	328,016	0.92	2015 vs. 2017	53.85	1	** < 0.0001**
F30–F39								
2015	1869	114,874	116,743	1.60	2015 vs. 2016	1.989	1	< 0.1584
2016	2034	119,447	121,481	1.67	2016 vs. 2017	17.40	1	** < 0.0001**
2017	2199	113,461	115,660	1.90	2015 vs. 2017	30.47	1	** < 0.0001**
F20–F29								
2015	609	49,465	50,074	1.22	2015 vs. 2016	6.560	1	< 0.0104
2016	730	51,476	52,206	1.40	2016 vs. 2017	2.711	1	< 0.0995
2017	745	48,185	48,930	1.52	2015 vs. 2017	17.22	1	** < 0.0001**

#### ECT and severity of major depression from January 2015 to December 2017

**Fig. 2:**
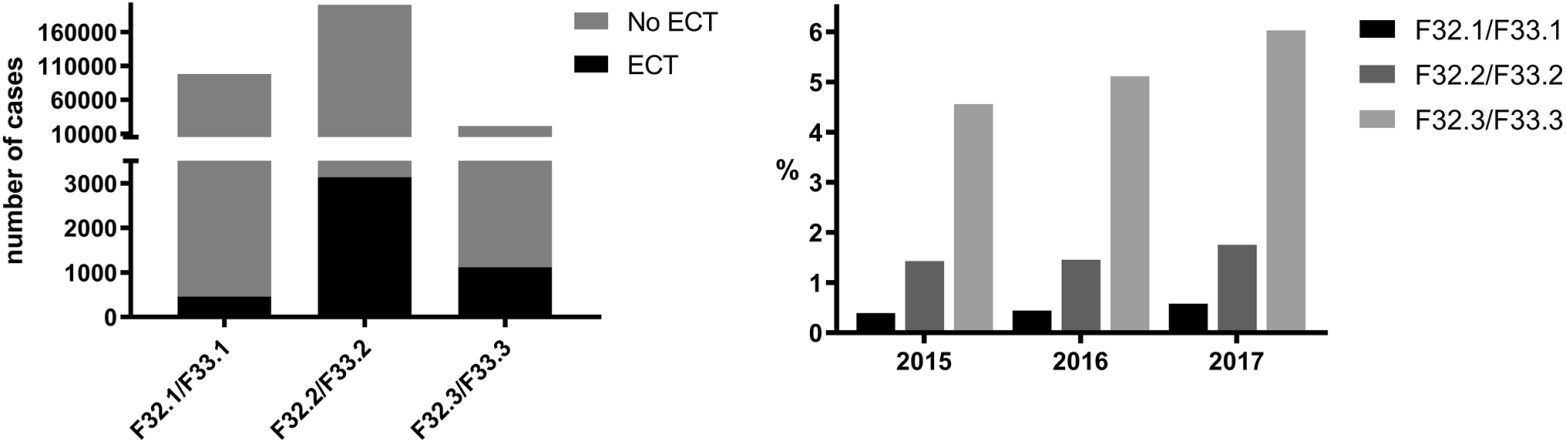
Total number of cases with and without performed ECT from January 2015 to December 2017 (left side) and annual proportions of ECT cases (%) (right side) considering the severity grade of major depression

The biggest share of cases associated with ECT was found in major depression with psychotic features (5.23%), followed by severe (1.55%) and moderate depressive episodes (0.47%). The differences were statistically significant with respect to F32.1/F33.1 vs. F32.2/F33.2 (*χ*^2^ 649.6, *p* < 0.0001), F32.2/F33.2 vs. F32.3/F33.3 (*χ*^2^ 1412, *p* < 0.0001) and F32.1/F33.1 vs. F32.3/F33.3 (*χ*^2^ 3053, *p* < 0.0001). In 2017, ECT was performed in 6.03% of all cases with psychotic depressions. For details see Fig. 2. As outlined previously, the shares of cases with performed ECT considering the year 2017 could be overrated due to the smaller database (see Table 1).

#### Maintenance and acute ECT from January 2015 to December 2017

**Fig. 3:**
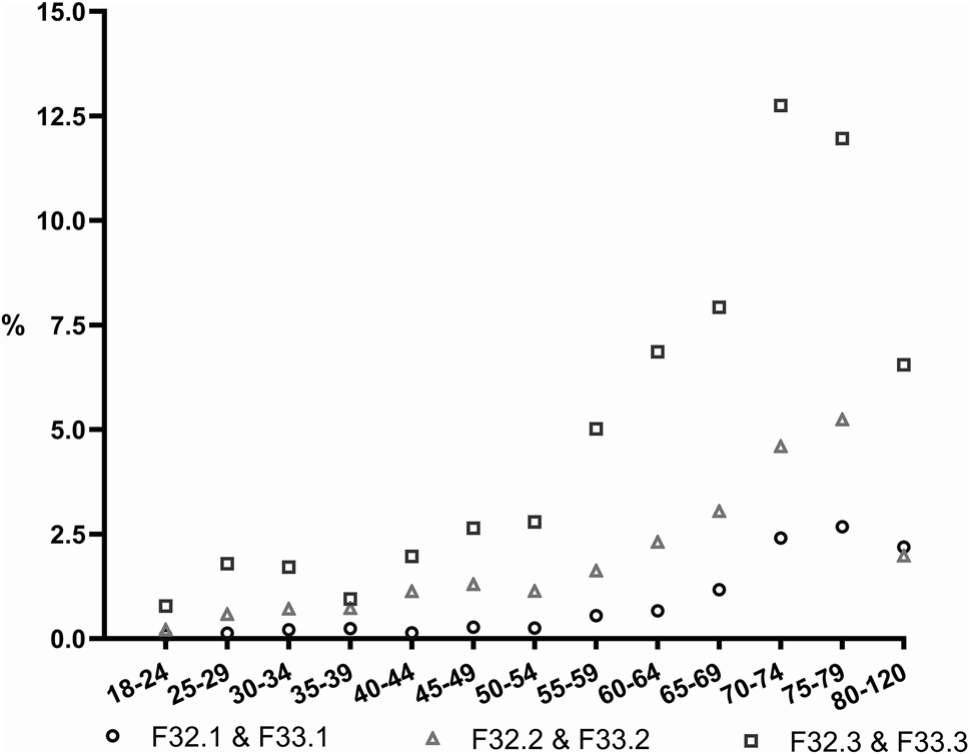
The proportion of ECT cases (in %) in terms of severity of depression and age

A total of 3524 cases (F00–F99) receiving ECT were short-term hospitalizations (≤ 4 days) corresponding to a rate of 42.1%. Maintenance ECT was performed in 837 cases with a diagnosis of psychosis (F20–F29). This corresponds to a rate of 40.2%. Regarding affective disorders, 2645 cases, corresponding to a rate of 43.3%, fulfilled the criterion for maintenance ECT (Fig. 3). Maintenance ECT was delivered in 21 cases in day- care settings corresponding to a share of 0.60% in terms of all maintenance ECT cases.

#### Comparison of age and gender in ECT cases from January 2015 to December 2017

Considering the main diagnoses F00–F99, the share of cases with performed ECT was significantly higher in an older (≥ 65 years) population compared to a younger (18–64 years) population (2.08% vs. 0.60%; *χ*^2^ 3570, *p* < 0.0001). This difference was also significant with respect to affective disorders (4.72% vs 1.10%; *χ*^2^ 3907, *p* < 0.0001) and psychosis diagnoses (2.14% vs. 1.30%; *χ*^2^ 67.70, *p* < 0.0001).

Interestingly, the proportion of ECT cases in major depression depended on both, age and severity of depression. The higher the severity grade of depression and the patients´ age, the higher was the share of cases with performed ECT (Fig. 3).

Considering F00–F99 diagnoses, 5482 patients were female and 2887 patients were male. This corresponds to a share of 1.14% females and 0.54% males, respectively. The difference was found to be significant in chi- square test (*χ*^2^ 1099, *p* < 0.0001).

A significant difference was also seen considering the group with affective disorders, F30-F39 (4064 (1.92%) female cases vs. 2038 (1.44%) male cases; *χ*^2^ 115.9, *p* < 0.0001). It is noteworthy that even more female cases were treated with ECT compared to male subjects if the main diagnosis was a psychosis (1303 (1.87%) female cases vs. 781 (0.96%); *χ*^2^ 231.1, *p* < 0.0001).

### Repetitive transcranial magnetic stimulation

#### Frequency of rTMS from January 2015 to December 2017

rTMS was carried out in 809 cases (91 in day-care and 718 in inpatient settings) between January 2015 and December 2017 corresponding to a share of 0.08% regarding the main diagnoses F00–F99. The majority (750 cases, 0.21% of all F30–F39 cases) of rTMS treatments was performed in affective disorders (F30–F39), mainly in major depression (701 cases, 0.22% of all F32 and F33 cases). rTMS was performed in 574 cases with severe major depression (0.27% of all F32.2 and F33.2 cases).

**Fig. 4:**
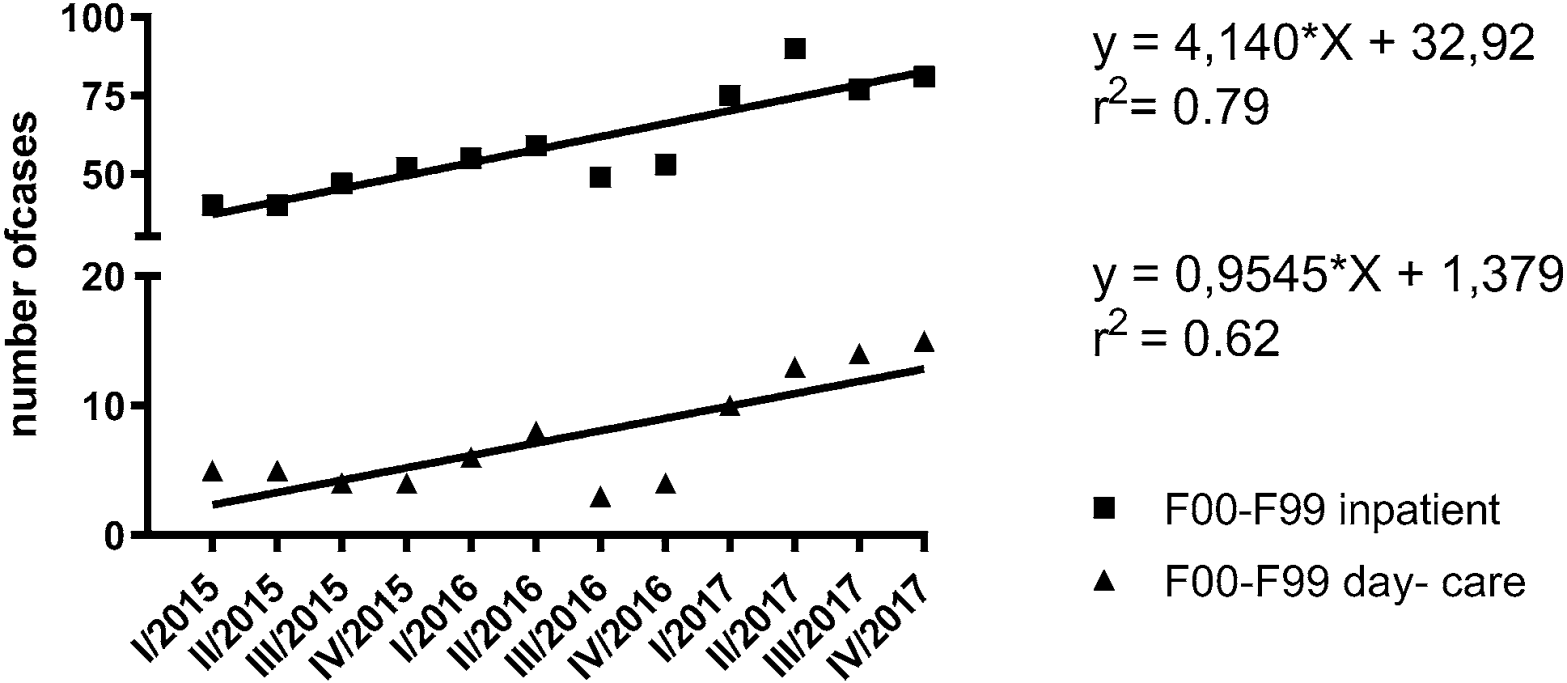
(Left side) Frequency of rTMS cases from January 2015 to December 2017 in inpatient and day- care settings considering F00–F99 diagnoses; Number of cases (*y* axis) are plotted per quarter (*x* axis); (right side) Linear regression analysis: equation of best-fit line, goodness-of-fit expressed in *r*^2^

As given in Fig. 4, the total number of cases with rTMS gradually increased between January 2015 and December 2017. Interestingly, rTMS was also increasingly carried out in day-care settings. The linear regression model suggests a high relationship (*r*^2^) between the number of cases in inpatient settings and observation period and even a moderate relationship considering cases in day- care settings. The slope was significant different than zero regarding inpatient settings (*F* 38.73, DFn 1.000, DFd 10.00, *p* < 0.0001) and day-care settings (*F* 17.01, DFn 1.000, DFd 10.00, *p* = 0.0021), respectively.

#### Frequency of rTMS considering main diagnoses from January 2015 to December 2017

In 2017, the overall number of cases with rTMS significantly increased compared to 2015 and 2016. Though rates were generally very low. The frequency of rTMS cases in 2015 did not significantly differ from that seen in 2016. In affective disorders (F30–F39), the proportion of cases increased in 2017 compared to 2015 and 2016. rTMS was primarily performed in severe major depressions (F32.2 and F33.2). For details see Table 3. As already outlined with respect to ECT cases, it should be taken into account that the results of statistical analyses shown in Table 3 could be partly influenced by the smaller case database of the year 2017 and did not necessarily reflect a higher relative use of rTMS in 2017.

**Table 3:** Comparison of the groups with and without rTMS between January 2015 and December 2017. Cases with the ICD-10 diagnoses F00–F99 (upper section), F30–F39 (middle section) and F32.2 and F33.2 (bottom section) were analyzed. Significant results are highlighted in bold characters

Year	rTMS	No rTMS	Total	rTMS (%)		*χ*^2^	*df*	*p* value
F00–F99								
2015	197	335,146	335,343	0.06	2015 vs. 2016	2.212	1	< 0.1370
2016	237	349,365	349,602	0.07	2016 vs. 2017	40.61	1	** < 0.0001**
2017	375	327,641	328,016	0.11	2015 vs. 2017	59.45	1	** < 0.0001**
F30–F39								
2015	174	116,569	116,743	0.15	2015 vs. 2016	4.459	1	< 0.0347
2016	224	121,257	121,481	0.18	2016 vs. 2017	35.18	1	** < 0.0001**
2017	352	115,308	115,660	0.30	2015 vs. 2017	62.05	1	** < 0.0001**
F32.2 and F33.2								
2015	143	65,636	65,779	0.22	2015 vs. 2016	2.691	1	< 0.1009
2016	182	69,547	69,729	0.26	2016 vs. 2017	25.173	1	** < 0**.**0004**
2017	249	67,316	67,565	0.37	2015 vs. 2017	25.98	1	** < 0.0001**

#### Comparison of age and gender in rTMS cases from January 2015 to December 2017

Considering the main diagnoses F00–F99 and F30–F39, the shares of rTMS cases were found to be higher in an older (≥ 65 years) population compared to a younger (18–64 years) population. The difference was statistically significant (F00–F99: 0.11% ≥ 65 years vs. 0.07% 18–64 years; *χ*^2^ 18.26, *p* < 0.0001; F30–F39: 0.27% ≥ 65 years vs. 0.20% 18–64 years; *χ*^2^ 10.75, *p* = 0.001). The proportions of rTMS performances in severe depression disorders did not significantly differ between both age groups (F32.2 + F33.2; 0.34% ≥ 65 years vs. 0.27% 18–64 years; *χ*^2^ 6.209, *p* = 0.0127).

Considering all psychiatric diagnoses, the use of rTMS was more likely in female patients (444 corresponding to 0.09%) compared to male patients (365 corresponding to 0.07%). With respect to the subgroup with severe major depression (F32.2 and F33.2) and affective disorders (F30–F39) the shares of rTMS cases among males were higher (0.33%, 270 cases and 0.24%, 340 cases) compared to the shares among females (0.25%, 304 cases and 0.19%, 410 cases). This was due to the lower prevalence of affective disorders in the male cohort. Differences in gender were found to be statistically significant with respect to F00–F99 and F32.2 and F33.2. (F00–F99, *χ*^2^ 17.47, *p* < 0.0001; F30–F39, *χ*^2^ 8.585, 1, *p* = 0.0034; F32.2 and F33.2; *χ*^2^ 12.06, *p* = 0.0005).

## Discussion

The present study evaluated the use of ECT and rTMS over a period of three years on the basis of a German wide, case-oriented psychiatric database. As outlined previously, less than one third of all clinics performing ECT contributed data for this study. The database is even smaller considering hospitals offering rTMS treatments. We found that ECT and rTMS are performed in inpatient and day- care settings in the German mental healthcare system. The number of cases with rTMS and ECT treatments appeared to increase between 2015 and 2017 as well as their shares. As shown in Table 1, this could be due to facilities introducing ECT and rTMS in the meanwhile but also to an increased treatment frequency of facilities which already established these stimulation therapies. However, it is important to mention that the proportions of rTMS and ECT cases considering the evaluation year 2017 could be overrated as the overall number of hospitals contributing data in 2017 was generally lower compared to the previous time periods.

### Current state of application of rTMS

In the present study rTMS was rarely used and mainly carried out in depressive disorders (701 cases corresponding to 86.7% of all rTMS cases). Epidemiological data on the use of rTMS in Germany are sparse. Previous results of a survey in 2015 about the use of rTMS in Germany showed that about 3400 inpatients were annually treated with rTMS in psychiatric hospitals [28]. The authors described that 1462 would receive rTMS for unipolar depression corresponding to roughly 43% of all psychiatric indications. 41% of surveyed psychiatric clinics reported a use of rTMS. In this study, about 5% of all clinics reported rTMS treatments in 2015. The difference could be due to a reporting bias as the survey study by Bürger et al. showed a quite low participation (response rate of 16%). Here, an overrepresentation of clinics using rTMS is possible as facilities applying rTMS were more likely to participate in the study. On the other hand, it might be possible that rTMS procedures were not consistently encoded in our database as rTMS is still not generally eligible for reimbursement. Therefore, the use of rTMS in this study is likely underrated.

### Current state of application of ECT

Prudic et al. [29] estimated about one million patients being treated with ECT worldwide every year. The proportion of patients receiving ECT in the USA was estimated to be 4.9 patients per 10.000 residents per year [30]. A recent meta-analysis found a lower prevalence of ECT in an inpatient cohort in the USA (0.4–1.3%) compared to other countries. For example, the prevalence of ECT ranged from 1.7 to 5.3% among an inpatient population for Scandinavian countries and was about 4% for a specialized German University Centre in 2002, respectively [31]. Similar to our data, the most frequent main diagnoses in the USA were affective disorders (unipolar/bipolar), ranging up to a proportion of 92% and psychosis were less mentioned (up to 29%) [29, 32–35]. Several epidemiological studies consistently described a decline in ECT delivery in the USA [36, 37] and state an undersupply of ECT [38]. Slade et al. [39] showed a small rate of ECT delivery (1.5%) among inpatients suffering from severe affective disorders.

Loh et al., reported that the use of ECT in Germany doubled in 2008 compared to previous data [24, 25, 40].The same workgroup estimated that 0.4 ‰ of all patients with depression and 1% of all inpatients with depression received ECT, respectively. In our study, the proportion of ECT cases was between 1.5% and > 5% for severe depressive disorders. Indeed, in our study the total number of ECT cases increased from 2015 to 2017. However, the proportions of cases treated with ECT appear quite low considering the high prevalence of treatment resistant depressions.

### Comparison of inpatient and day-care setting

rTMS was more often performed in day- care settings compared to ECT. Unfortunately, our database does not comprise ambulatory rTMS services. Indeed, rTMS is feasible in day-care and ambulatory settings as its clinical tolerability is acceptable [8]. The trend of increasing inpatient and day-care cases treated with rTMS is quite interesting and needs to be investigated in further studies addressing the question if this reflects a growing demand for rTMS treatments. Indeed, we recognized an increasing demand for that stimulation technique in our clinic.

Another important finding of the study was that ECT was rarely delivered in day- care settings. This could be due to a generally higher need for surveillance after treatment compared to rTMS. Furthermore, patients receiving acute ECT commonly suffer from a higher severity of illness, i.e. suicidal ideation and delusions. But ambulatory ECT can be possible and safe for suitable patients [41, 42]. In Canada, it was reported that 90% of ECT treatments were delivered on an outpatient basis [43]. Loh et al. [24] reported 79 hospitals performing maintenance ECT and that only 9% of these delivered maintenance ECT on an outpatient basis. Unfortunately, our database did not comprise information on the current ambulatory ECT services.

### Current state of maintenance ECT

Maintenance ECT reduces the risk of relapses, especially considering mood disorders [44]. In this context, we found a high proportion of (> 40%) short-term hospitalization (≤ 4 days) which we considered as maintenance ECT cases. Information about the total amount of ECT treatments and the corresponding indications (maintenance and acute ECT treatments) were not directly available due to the case-based structure of the database. Therefore, this case-based analysis can be misleading giving the impression that it contains a high number of ECT treatment sessions. To date, only about 15% of ECT treatments are supposed to be maintenance treatments in Germany. However, it is noteworthy that the vast number of these patients (99.40% of all ECT cases with short-term hospitalizations) received a regular inpatient care. In the presence of increasing financial restrictions and limited psychiatric inpatient capacity it is of great interest to identify appropriate candidates for ambulatory or day-care ECT services. Future studies combining clinical and routine data might address this issue.

### Age and gender in the ECT cohort

The higher proportion of ECT in elderly and among females was frequently shown in previous studies [31], especially considering ECT in depressive disorders [45]. In Europe, the mean age ranged between 49–66 years [31]. The findings of previous studies in the USA were similar in terms of gender (66–79% were female) and age (48–59% over 60 years), respectively [29, 33–35, 46–48]. Loh et al. [24] reported a higher proportion of females (57%) and a lower proportion of patients under 50 years (41%) for Germany in 2008. ECT is effective in late-life depression [17, 49] and the course of late-life depression is often complicated due to chronicity, recurrences, poor drug tolerability [50–52] and limited effectiveness of psychopharmacotherapy [53]. Greater age and severity of depressive symptoms were shown to be, albeit weak, clinical predictors of higher response to ECT which stands in line with the higher use of ECT among elderly in the present study [54]. Consequently, ECT should always be considered as a treatment option in late-life depression.

### Age and gender in the rTMS cohort

Geriatric age was associated with a higher proportion of rTMS cases considering main diagnoses F00-F99 and affective disorders. For major depression, it seems that higher age favors rTMS but the difference between the young and old age group was not statistically significant. Age is controversially discussed as a predictor for response to rTMS. Previous studies showed better efficacy of rTMS in younger patients with depression [55–58]. Indeed, it is supposed that cortical atrophy of the frontal lobe and higher rate of somatic symptoms are reasons for the fewer efficacy of rTMS in late-life depression [57, 59]. Nevertheless, an accelerated rTMS protocol was shown to be more efficient in older depressed people (> 60 years) [60]. Recently, a trial with deep-rTMS provided promising results [61].

With except of the main diagnoses F00–F99 the share of rTMS cases among males was higher compared to the share of rTMS cases among females due to a lower prevalence of major depression among males. Eventually, there is no convincing evidence for gender as a feasible predictor for response to rTMS in depression [62–64]. Future investigations should address the role of gender in rTMS treatment in depression.

### Strength and limitations

The large number of cases, exceeding 1 Million, is the major strength of this study. All diagnostic data were assessed by medical professionals in day-care and inpatient settings and, therefore, offer a high external validity. In contrast to survey studies there are no concerns on a reporting bias with respect to ECT codes as these codes are mandatory during the reimbursement process. The present study had multiple limitations that need to be considered. First limitation is that the presented database was collected from hospitals on a voluntary basis and does not reflect an official governmental report. Compared to the annually reported data of the Federal Office of Statistics of Germany, this study database comprises approximately one third of all registered psychiatric cases. Although a large part of hospitals joined this project, the database is not necessarily representative for the entire German healthcare system. The second limitation is that patient-level data are not accessible due to German and the European Union’s data protection laws. The analyzed routine data did not provide detailed information about the patient´s clinical status as German hospital routine data were primarily collected for reimbursement purposes. Furthermore, information about the absolute number of ECT and rTMS treatments per case were not directly accessible due to the case- based data structure. A further important limitation is the short observation period. Next limitation is a possible selection bias since patients with low severity grade are more likely to be treated in day-care settings compared to severe affected patients being more likely to be treated with ECT in inpatient settings. Furthermore, the analysis depends on the accuracy of assessing procedural codes (OPS). As rTMS is not regularly eligible for reimbursement in contrast to ECT it could be possible that the professionals do not consistently encoded for rTMS (reporting bias). Additionally, rTMS is also delivered on an outpatient basis. Therefore, the database probably underestimates the current use of rTMS in German psychiatry. Also, ECT is performed on an outpatient basis in some cases, especially maintenance ECT.

## Conclusions

The present study based upon a German sample of > 1.000.000 psychiatric cases. The typical psychiatric case treated with ECT was female, of old age and depressed. The higher the severity grade of major depression and age, the higher was the share of ECT cases. Repetitive TMS was primarily performed in depressed patients.

In this study, ECT and rTMS cases increased annually considering the period between 2015 and 2017. Furthermore, ECT and rTMS were hardly applied in day- care settings. The trends found in this study certainly need to be validated in future investigations. Nevertheless, we showed a low use of ECT which stands in contrast to the high prevalence of treatment resistant depression (> 30%) and available local guidelines. An increasing demand for ECT will be a challenge and may require a shift to more ambulatory or day-care services due to capacity limits and economic issues. In this context, future studies combining clinical and routine data could be of great interest as we showed a majority of short- term inpatients possibly receiving maintenance ECT. To extend ambulatory ECT services, it is however important to establish appropriate financial conditions as there is still no reimbursement for outpatient ECT in Germany.
