# Correction to: Current clinical practice of electroconvulsive therapy and repetitive transcranial magnetic stimulation in psychiatry, a German sample

**DOI:** 10.1007/s00406-021-01275-7

**Published:** 2021-06-05

**Authors:** Charles Timäus, Jonathan Vogelgsang, Bernhard Kis, Katrin Radenbach, Claus Wolff-Menzler, Kiriaki Mavridou, Stephan Gyßer, Philipp Hessmann, Jens Wiltfang

**Affiliations:** 1 Department of Psychiatry and Psychotherapy, University Medical Center Göttingen, University of Göttingen, Von-Siebold-Str. 5, 37075 Göttingen, Germany; 2 Division of Software Development and Business Intelligence, GSG Consulting GmbH, Flughafenring 2, 44319 Dortmund, Germany; 3 German Center for Neurodegenerative Diseases (DZNE), Von-Siebold-Str. 3a, 37075 Göttingen, Germany; 4 iBiMED, Medical Science Department, University of Aveiro, Aveiro, Portugal; 5 Department of Psychiatry, Psychotherapy and Psychosomatics, Contilia Group, St. Elisabeth Hospital, Hattingen, Germany

## Correction to: European Archives of Psychiatry and Clinical Neuroscience (2021) 271:181–190 10.1007/s00406-020-01099-x

The article “Current clinical practice of electroconvulsive therapy and repetitive transcranial magnetic stimulation in psychiatry, a German sample” written by Charles Timäus, Jonathan Vogelgsang, Bernhard Kis, Katrin Radenbach, Claus Wolf-Menzler, Kiriaki Mavridou, Stephan Gyßer, Philipp Hessmann and Jens Wiltfang was originally published electronically on the publisher’s internet portal on January 29, 2020 without open access. With the author(s)’ decision to opt for Open Choice the copyright of the article changed to © The Author(s) 2021 and the article is forthwith distributed under a Creative Commons Attribution 4.0 International License, which permits use, sharing, adaptation, distribution and reproduction in any medium or format, as long as you give appropriate credit to the original author(s) and the source, provide a link to the Creative Commons licence, and indicate if changes were made. The images or other third-party material in this article are included in the article’s Creative Commons licence, unless indicated otherwise in a credit line to the material. If material is not included in the article’s Creative Commons licence and your intended use is not permitted by statutory regulation or exceeds the permitted use, you will need to obtain permission directly from the copyright holder. To view a copy of this licence, visit http://creativecommons.org/licenses/by/4.0/. Open Access funding enabled and organized by Projekt DEAL.

